# An Intelligent Fault Diagnosis Based on Adversarial Generating Module and Semi-supervised Convolutional Neural Network

**DOI:** 10.1155/2022/1679836

**Published:** 2022-06-24

**Authors:** Qing Ye, Changhua Liu

**Affiliations:** ^1^School of Computer Science, Yangtze University, Jingzhou 430023, China; ^2^General Office, Yangtze University, Jingzhou 430023, China

## Abstract

Aiming at the existing problems in machinery monitoring data such as high cost of labeling and lack of typical failure samples, this paper launches a research on the semi-supervised-style intelligent fault diagnosis. Taking a great mount of unlabeled data and only a small quantity of labeled data as inputs, a novel fault diagnosis framework based on adversarial generating module and semi-supervised convolutional neural network (SSCNN) is proposed. Firstly, a semi-supervised learning module based on manifold-regularization-based fuzzy clustering discrimination (MRFCD) is proposed to make full use of the valuable fault-related information contained in unlabeled data. Secondly, MRFCD was introduced into CNN to construct pseudo-labels and estimate the objective function of unlabeled data. Then, the semi-supervised deep-learning-module-based MRFCD-SSCNN is established. Thirdly, to enhance the effect of MRFCD-SSCNN, generative adversarial network (GAN) was utilized to increase the size of training data under failure conditions. The framework based on GAN-MRFCD-SSCNN is proposed to achieve semi-supervised style intelligent fault diagnosis. To verify the performance of the diagnostic framework, vibrational signals of main reducer collected from actual test rig are employed. The comparative results confirm that the proposed framework outperforms some classical semi-supervised diagnostic models and achieves the accuracy of 96.2% using only 400 labeled samples.

## 1. Introduction

Main reducer is an important component in rear axle of automobile, and the operating conditions of main reducer directly affect the safety and comfort during running of automobile [1–3]. Therefore, to avoid catastrophic accidents and to improve the reliability of automobile, intelligent recognition of failure patterns for main reducer should be further researched deeply [4].

With the success of deep learning in the fields of image recognition and speech recognition, more and more scholars have applied it into intelligent machinery fault diagnosis and achieved expected results [5, 6]. Taking stacked auto-encoder (SAE) [7, 8], convolutional neural networks (CNN) [9, 10], and deep neural networks (DNN) [11, 12], as examples, the advantages of deep learning mainly lie in the feature representation capability of its multi-layer mapping network structure and the feature extraction ability in processing extensive condition monitoring data [13, 14]. Among these models, CNN is the most commonly applied in intelligent fault diagnosis based on vibration signals [15]. Luyang Jing utilized convolutional neural network to extract typical features of gearbox [16]. Long Wen introduced convolutional neural network into the failure pattern recognition of pump and gear [17]. Janssens utilized CNN to improve the accuracy of fault detection for rolling bearing [18]. Qing Ye constructed a parallel convolutional auto-encoder-based framework to actualize fault diagnosis of main reducer [19]. G Jiang proposed a novel diagnostic model based on multi-scale CNN to realize fault diagnosis of turbine gearbox [20]. Chen employed deep CNN to identify failures of planetary gearbox based on vibrational signal data [21].

The diagnostic models based on deep learning model in above literature require a balanced monitoring data containing a large number of labeled training data [22]. Additionally, training data should be collected under both the normal state and the fault states. Actually, there is a typical bottleneck in real situation of machinery fault diagnosis. The labeled data are difficult to obtain because labeling is a costly and time-consuming process. To effectively tackle the problem of low data availability, semi-supervised learning based on extreme unbalanced combination of labeled data and unlabeled data is introduced into deep learning model [23–25]. Yue et al. combined CNN with semi-supervised learning to recognize radar images [26]. Rasmus et al. put forward a semi-supervised learning method on the basis of ladder network [27]. For models that combine semi-supervised learning and deep learning, how to measure the loss function of unlabeled data is a pivotal issue which would obviously affect the utilization of unlabeled data and performance of the model.

These semi-supervised deep learning models achieved better results by extracting representative features from both plentiful labeled data and unlabeled data. However, there are still some areas for optimization and improvement such as how to fully reduce the imbalance of data and to enhance the value density. Among the massive monitoring data obtained through signal acquisition, the monitoring data under normal state accounts for the vast majority while the monitoring data under fault states is obviously insufficient [28]. Some intelligent algorithms can be used to extend dataset under multiple different conditions so as to solve the problem of low value density. Generative adversarial network (GAN) which is inspired from two-person zero-sum game in game theory was proposed by Goodfellow in 2014 [29]. In the area of picture synthesis, GAN is widely used and has achieved great success [30]. Zhedong Zheng et al. utilized DCGAN to generate abundant unlabeled images and inputted into deep learning model based on CNN to improve the baseline of person re-identification [31]. Ashish Shrivastava et al. adopted a modified adversarial network based on GAN to reduce the gap between synthetic images and real images, and the generated images can achieve excellent results in the training of the hand pose estimation model [32]. In fault diagnosis of machinery, GAN can be utilized to generate signals under multiple different conditions so as to solve the difficulties of lacking typical fault type samples [33, 34].

To tackle the abovementioned problems, a semi-supervised style intelligent fault diagnosis framework based on GAN and CNN is proposed and applied into failure patterns recognition of the main reducer. The contributions of the proposed framework are categorized as follows:

1. with the purpose of fully utilizing plenty of unlabeled data during the model training, we take the clustering properties of data distribution manifold structure into account and improve the traditional manifold regularization. Then, we propose a semi-supervised learning module based on manifold regularization and fuzzy clustering discrimination (MRFCD) module.
2. The proposed MRFCD module is introduced into convolutional neural network to construct the pseudo-labels of the loss function unlabeled data, and the semi-supervised deep learning model based on MRFCD-SSCNN is established.
3. In order to enhance the performance of MRFCD-SSCNN model, GAN is utilized to expand the training set size under typical fault conditions. The framework based on GAN-MRFCD-SSCNN is presented to actualize high accuracy of fault identification of main reducer by using vibrational signals.

The rest content of this paper is organized in the following four parts. In Section 2, the fundamental theories of generative adversarial network and convolutional neural network are given. In Section 3, the proposed diagnostic framework is described in detail. In Section 4, the proposed model is evaluated by using vibration signals collected from test rig. In Section 5, a conclusion is given.

## 2. The Fundamental Theories

### 2.1. Generative Adversarial Network (GAN)

**Figure 1:**
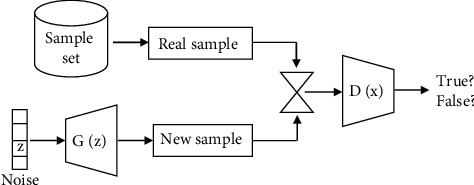
Generative adversarial network.

As a novel generative model, the objective of GAN is to produce new samples with the same characteristics as the training samples. As shown in Figure 1, GAN contains two free-running modules: a generator (*G*) and a discriminator (*D*). The task of generator is to produce new samples under the control of certain hidden variables, and the task of discriminator is to discriminate the samples produced by generator and the real training samples. During the process of adversarial training, each network is trained on the basis of fixing another network [35].

Given an arbitrary random vector *z*, the generator network *G*(*z*) produces a new sample. For a real sample *x*, the discriminator neural network *D*(*x*) takes *x* and a generated sample *G*(*z*) as input, and the output determines whether the input is a real sample or a generated sample by using the Sigmoid function.

The training process based on batch stochastic gradient descent is to maximize the objective function for the discriminator and to minimize the discriminator function for the generator. The total loss function is described as follows:

(1) $$\begin{array}{c} V\left(D,G\right)=E_{x∼p_{da\;ta}\left(x\right)}\left[\log\;D\left(x\right)\right]+E_{z∼p_{z}\left(z\right)}\left[\log\left(1-D\left(G\left(z\right)\right.\right)\right] \end{array}$$

in which *z* denotes Gaussian noise inputted into the generator, *G*(*z*) denotes the output of the generator, *p*_*data*_ denotes the probability distribution of the real samples, *p*_*z*_ denotes the probability distribution of the added noise, and *D*(*x*) denotes the discriminating results of the discriminator. Parameters of generator network and discriminator network are trained using training samples with the objection as follows:

(2) $$\begin{array}{c} \underset{G}{\min}\underset{D}{\max}V\left.\left(D,G\right)\right] \end{array}$$

### 2.2. Convolutional Neural Network (CNN)

In contrast to several traditional shallow neural networks, convolutional neural network with complex structure contains an input layer, multiple convolutional layers and pooling layers, multiple fully connected layers and an output layer [36, 37]. Each neuron in the convolutional layer only extracted local information from one receptive field in the previous layer, and the neurons weighted summed the information within the receptive field to obtain feature maps. The feature maps are downsampled by pooling layers. After multiple convolutional-pooling layers, one fully connected layer maps the extracted representative features to a class space. When the recognition target is multi-class, a softmax classifier is usually used as the output layer of the fully connected layer.

Though convolutional neural network has many layers, information transmission is still feedforward from input layer to output layer. By calculating the training error of final output layer, back propagate the gradient of error layer by layer to update the parameters of the previous layers [38, 39].

## 3. Proposed Methodology

### 3.1. Design of Manifold-Based Fuzzy Clustering Discrimination Regularization (MFCDR)

Suppose that a multi-class sample set contains *m* labeled samples {(*x*_*i*_, *y*_*i*_)}_*i*=1,2,…,*m*_ and *n* unlabeled samples {(*x*_*j*_)}_*j*=1,…,*n*_, in which *x*_*i*_ and *x*_*j*_ represent feature vectors, *y*_*i*_ represents the label of samples, *k* denotes the number of classes, and *m* is far less than *n*.

To make full use of the manifold structure hidden in a great mount of unlabeled samples, the entire sample set is divided into *C* fuzzy clusters by using the fuzzy clustering method [40, 41], then, each sample is assigned a fuzzy clustering label to obtain the clustering discrimination information of the sample set. Combine the fuzzy clustering labels *t*_*j*_^*c*^ of each sample into a clustering vector *T*^*C*^=[*t*_1_^*c*^,…, *t*_*m*+*n*_^*c*^], in which *t*_*j*_^*c*^∈[1, *C*]. Considering the reliability of the clustering results, a membership grade vector is defined as: *M*=[*m*_1_^*c*^,…, *m*_*m*+*n*_^*c*^], in which *m*_*j*_^*c*^ measure the membership degree that the *j*th sample belong to the *c*th fuzzy cluster. *m*_*j*_^*c*^ is defined as follows:

(3) $$\begin{array}{c} m_{j}^{c}=\begin{array}{cc} 1 & if\;x_{j}\;is\;unlabele\;d\;sample,\;\\ \mu & \;others, \end{array}\mu \in \left[0,1\right], \end{array}$$

in which *μ* varies inversely to the gap of each sample against the cluster's central point after fuzzy clustering. In order to measure the dependability of obtained clustering results, we define a matrix *R* = *MM*^T^ based on the membership degree vector. The clustering discrimination matrix of the sample set *S*^*c*^ is determined by the clustering label and the clustering reliability matrix *R*:

(4) $$\begin{array}{c} S_{ij}^{c}=\left\{\begin{array}{c} 1,\;if\;t_{i}^{c}=t_{j}^{c}\\ 1,\;if\;t_{i}^{c}\neq t_{j}^{c}\;an\;d\;R_{ij}\leq 0.5,\\ -1,\;if\;t_{i}^{c}\neq t_{j}^{c}\;an\;d\;R_{ij}>0.5,\\ i,j=1,\ldots \left(m+n\right), \end{array}\right. \end{array}$$

in which 0.5 is the frequently-used threshold and *S*^*c*^ ∈ R^(*m*+*n*)×(*m*+*n*)^ [42]. For *m* labeled samples, in order to retain their real labels, a label discrimination matrix *S*^*m*^ was generated as follows:

(5) $$\begin{array}{c} S_{ij}^{m}=\left\{\begin{array}{cc} 1 & ify_{i}=y_{j},\\ -1 & \;ify_{i}\neq y_{j}, \end{array}\right.\;i,j=1,\ldots ,m \end{array}$$

Above all, the final clustering discrimination matrix *S* ∈ R^(*m*+*n*)×(*m*+*n*)^ is jointly determined by *S*^*c*^ and *S*^*m*^:

(6) $$\begin{array}{c} S_{ij}=\left\{\begin{array}{cc} S_{ij}^{m} & \text{if }x_{i\;},x_{j}\;are\;both\;labele\;d\;sample,\\ S_{ij}^{c} & others, \end{array}i,j=1,\ldots \left(m+n\right)\right. \end{array}$$

Thus, the optimal solution of fuzzy clustering discrimination using a large number of unlabeled samples should satisfy a pair-constrained regularization that contains both the lower intra-class dispersion and the higher inter-class separation:

(7) $$\begin{array}{c} \underset{f}{\min}\frac{1}{2}\sum_{i,j=1}^{m+n}f\left(x_{i}\right)-f{\left(x_{j}\right)}^{2}W_{c,ij}-\frac{1}{2}\overset{m+n}{\underset{i,j=1}{\sum }}f\left(x_{i}\right)-f{\left(x_{j}\right)}^{2}W_{s,ij} \end{array}$$

in which *f*(·) represents the decision function of classification, *W*_*c*_ and *W*_*s*_ are separately the weight matrix of intra-class and inter-class as shown below:

(8) $$\begin{array}{c} W_{c,ij}=\left\{\begin{array}{cc} 1, & ifS_{ij}=1,\\ 0, & ifS_{ij}=-1, \end{array}\right. \end{array}$$

(9) $$\begin{array}{c} W_{s,ij}=\left\{\begin{array}{cc} 1, & ifS_{ij}=-1,\\ 0, & \;ifS_{ij}=1. \end{array}\right.. \end{array}$$

In this paper, a manifold-regularization-based fuzzy clustering discrimination framework (MRFCD) which is based on fuzzy clustering and manifold assumptions is proposed. That is, samples belonging to the same cluster with high clustering reliability are set to the same labels. By combining the fuzzy clustering discrimination of the entire sample set with manifold regularization, the objective function of the optimization problem is expressed as:

(10) $$\begin{array}{c} \underset{f}{\text{ min }}\frac{1}{l}\overset{m}{\underset{i=1}{\sum }}V\left(x_{i},y_{i},f\left(x_{i}\right)\right)+\gamma_{A}f{\;}_{K}^{2}+\frac{1}{2}\overset{m+n}{\underset{i,j=1}{\sum }}f\left(x_{i}\right)-f{\left(x_{j}\right)}^{2}W_{c,ij}\\ -\;\frac{1}{2}\overset{m+n}{\underset{i,j=1}{\sum }}f\left(x_{i}\right)-f{\left(x_{j}\right)}^{2}W_{s,ij}, \end{array}$$

in which *V*(·) represents the loss function of *f*(·), *γ*_*A*_ represents the tradeoff parameter. In formula (10), the first regularization item *f*_*K*_^2^ denotes the complexity of decision function in reproducing kernel Hilbert space (RKHS).

### 3.2. Design of MRFCD-SSCNN

The optimization problem of MRFCD can be seen as the loss function of semi-supervised model using both the labeled samples and the unlabeled samples. In the objective function of MRFCD, the first regularization item denotes the loss function of decision function by using *m* labeled samples, and the second regularization item is to restrict the complexity of decision function. Based on the proposed MRFCD module and convolutional neural network (CNN), we present a semi-supervised model based on MRFCD-SSCNN which can be trained by a combination of labeled samples and unlabeled samples.

In the proposed model, cross entropy is employed to define the loss function *V*(·) of *m* labeled samples in equation (10). The loss function of MRFCD-SSCNN is expressed as follows:

(11) $$\begin{array}{c} L\left(\theta \right)=L_{m}\left(\theta \right)+L_{m+n}\left(\theta \right), \end{array}$$

in which *θ* denotes the parameter of model; *L*_*m*+*n*_(*θ*) represents the loss function of the entire sample set and is the last two items in equation (10); and *L*_*m*_(*θ*) represents the loss function of *m* labeled samples.

**Figure 2:**
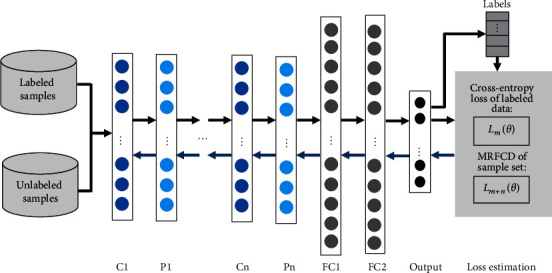
The structural representation of the semi-supervised model.

The architecture of the semi-supervised model based on MRFCD-SSCNN is as shown as Figure 2. As shown in Figure 2, the training process of deep neural network consists of two procedures: information forward propagation and error back propagation. The optimization problem is to minimize the loss function which is shown in equation (11):

(12) $$\begin{array}{c} \underset{\theta }{\min}\left(L_{m}\left(\theta \right)+L_{m+n}\left(\theta \right)\right) \end{array}$$

In the process of forward propagation, network weights are initialized randomly and the classification errors which can be used to measure the loss function are generated. In the process of back propagation, network weights are adjusted iteratively according to the optimization problem.

### 3.3. The Proposed Diagnostic Framework

Given the strong data generation effect of GAN, we employed this algorithm to generate samples under various working conditions to effectively solve the insufficient amount of typical fault type samples in monitoring data. Additionally, the semi-supervised model based on MRFCD-SSCNN can achieve preferable performance of classification without abundant labeled samples.

**Figure 3:**
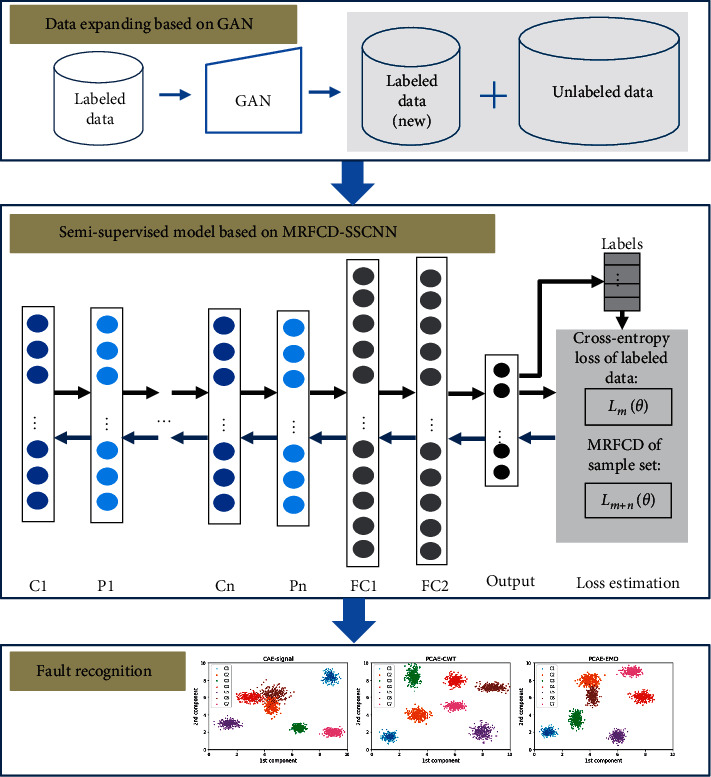
The proposed diagnostic framework.

This paper proposes a diagnostic framework based on GAN-MRFCD-SSCNN by combining the characteristics of abovementioned sample generation procedure and semi-supervised classification procedure together. As shown in Figure 3, the diagnostic framework can be described as follows:

1. Collect vibrational data from acceleration sensors under real conditions, and label tiny amounts of samples of known fault types.
2. For samples with different labels, new samples of the corresponding labels are generated using GAN to expand dataset and achieve the purpose of value density improvement.
3. The expanded datasets are segmented into two subsets: a training set and a testing set. The two subsets are composed of a combination of a great mount of unlabeled samples and a small number of labeled samples. Build CNN with initialized parameters.
4. The training set is utilized to actualize diagnostic framework training based on MRFCD-SSCNN, and obtain the loss function of labeled dataset and unlabeled dataset to measure the classification error by using the manifold structure and fuzzy clustering discrimination.
5. According to error gradient, update the network parameters of GAN-MRFCD-SSCNN-based framework to achieve the training of framework.
6. The testing set is utilized to test and estimate the diagnostic accuracy of the proposed framework.

## 4. Experiment and Discuss

### 4.1. Experimental Environment and Setup

**Figure 4:**
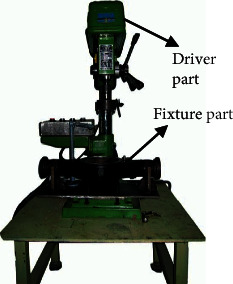
The test rig.

**Figure 5:**
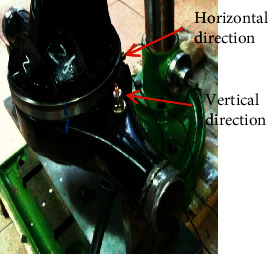
Sensor installation.

**Figure 6:**
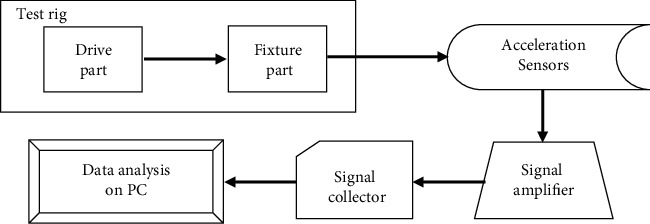
Experimental setup chart.

To validate the capability of the proposed diagnostic framework, a range of comparative analysis experiments are carried out. As shown in Figure 4, the experiments are set up on a real test rig which contains a driver part and a fixture part. The driver part is used to drive and control the rotation of the turntable on the fixture part. The turntable on the detection fixture is designed to simulate the actual working conditions of main reducer under a fixed rotating speed. As shown in Figure 5, two acceleration sensors are set up in horizontal and vertical direction of the device to collect vibrational signals which are the most frequently used in machinery diagnosis. The whole experimental setup is composed of the test rig, two acceleration sensors, a signal amplifier, a signal collector, and a PC. Due to the weakness of the collected vibrational signals, they are amplified by a signal amplifier and then fed into the computer to process and analysis. The experimental setup chart is in Figure 6.

According to the previous researches, the most common faults usually occur on the gear pair, such as gear crack, misalignment, gear interference, and gear hard point. We collect vibrational signals of 7 failure patterns and health pattern under the rotation speed of 1200 r/s to construct original dataset. The list of various patterns is as shown as Table 1. Considering the generality and stability of the collected vibrational signals, simulations are repeated for ten times for each pattern and signals in the most stable duration of 2 seconds are collected. The sampling frequency cannot be lower than the gear engagement frequency which is 12 kHz.

With the pre-processing of data, 500 samples of each pattern with the length of 1024 are obtained. 80% of the sample sets of each pattern is randomly selected to train the semi-supervised model. The remaining 20% of samples are employed to estimate the capability of the trained model. The training sets of 8 patterns are randomly divided into unlabeled dataset and labeled dataset, with 25 samples used as labeled dataset, and the rest of 375 samples are used as unlabeled samples by hiding the corresponding category markers. The distribution of dataset is shown in Table 2.

The data analysis and experiments are carried out on PC with 3.2 GHz and Intel i7 CPU, NVIDIA GeForce GTX 1080Ti GPU, and 32G RAM. We employed Tensorflow to build and train the deep-learning-based framework.

### 4.2. Data Expanding with GAN

Before training of the semi-supervised model based on MRFCD-SSCNN, GAN is utilized to expand the size of dataset. The network structure of generator and discriminator separately contain 4 convolutional layers and one fully connected layer. During the first procedure, the generator network inputs a 100-dimensional noise signal matrix conforming to the Gaussian distribution to obtain a 1024-dimensional generated signal matrix. Then, the generated signal matrix is merged with a 1024-dimensional real signal of a main reducer and fed into the discriminator network. With the activation function of sigmoid, discriminator network labels the real signal with 1 and labels the generated signal of noise with 0. Keeping the weights of the generator network constant, the weights of discriminator network are updated by BP algorithm based on the loss value.

In the second procedure, a 100-dimensional noise signal matrix conforming to the Gaussian distribution is input into the generator network, then, the output is a 1024-dimensional generated signal matrix which is entered into the discriminator network. The output matrix of the discriminator network with the fixed parameters is compared with the matrix with all the values of 1, and BP algorithm is employed to update the generator network parameters.

Using GAN, the sample size of each pattern is expanded from 500 to 2000, and the size of expanded dataset is 16000*∗*1024.

### 4.3. Architecture Design

With the expanded dataset, the semi-supervised diagnostic framework based on GAN-MRFCD-SSCNN can make full use of a great deal of samples without labels. The network structure of 1-D CNN contains deep feature extraction and fault classification. To achieve the extraction of deep features, three pairs of convolutional layers and down-sampling layers are arranged staggered. With activation function of ReLU, convolutional layers are utilized to transform input data into feature maps. Down-sampling layers are utilized to achieve feature dimension reduction. After that, two fully connected layers are arranged with the purpose of transferring the feature space into class space.

The architecture of the proposed framework is described in Table 3. The convolutional kernels of the three convolutional layers are 1*∗*16, 1*∗*6, and 1*∗*6. Additionally, the number of filters for each convolutional layer is twice as the upper layer. The three down-sampling layers adopt max-pooling strategy, and the pool size is 1*∗*2. To avoid over-fitting, dropout layer is added into the deep learning network.

In order to achieve fault classification, the feature maps obtained from feature extraction section are flattened and fed into following three fully connected layers. Then, the output of these layers is input into a softmax layer to obtain the decision vector for predicting the fault types of the main reducer.

Before training the proposed framework, some hyper-parameters are set to the optimal values in advance by using 10-fold cross-validation method as shown in Table 4.

### 4.4. Experimental Results and Comparative Analysis

#### 4.4.1. The Important Role of GAN

In the research, to reduce the imbalance of dataset and to enhance the value density, the size of the whole dataset is expanded from 4000 to 16000 by using GAN. To validate the effect of GAN in the diagnostic framework based on GAN-MRFCD-SSCNN, a range of contrastive experiments are carried out on the raw dataset and the expanded dataset. Under standard circumstances, 80% of the entire datasets are regarded as training set and the remaining 20% are regarded as testing set. In this way, only 400 samples in the expanded training set are used with their labels. For the rest 12400 samples, the corresponding labels are hidden.

**Figure 7:**
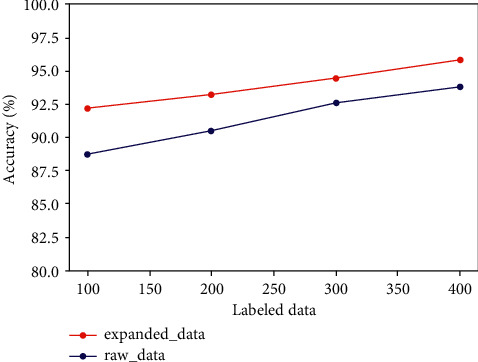
Contrastive results under the raw dataset and the expanded dataset.

The training set of raw dataset and expanded dataset are employed to train the semi-supervised diagnostic framework separately for performance comparison. Furthermore, to further measure the effect of different amounts of labeled data on diagnostic performance, we randomly selected 100, 200, 300, and 400 samples from the training set of raw dataset and expanded dataset as labeled samples. The contrastive results are shown in Figure 7.

As Figure 7 suggests, the overall diagnostic result of the semi-supervised framework based on MRFCD-SSCNN with raw dataset is obviously inferior to the expanded dataset. It proves that the amount of training set severely affects the diagnostic outcomes. For both the raw dataset and expanded dataset, the diagnostic results are improved as the number of labeled samples in the training set increases.

Regardless, the framework based on GAN-MRFCD-SSCNN can achieve excellent diagnostic effect even with a small quantity of labeled samples when using ample extended datasets as training set. The comparative results of this set of experiments all effectively demonstrate the important role of GAN in the proposed framework. The performance superiority of the framework can effectively solve the typical conundrum of data labeling difficulties in the field of mechanical engineering.

#### 4.4.2. The Effect of MRFCD

The proposed semi-supervised diagnostic model based on MRFCD-SSCNN relies on MRFCD to construct the pseudo-labels and to measure the loss function of data without labels, and then, to achieve semi-supervised learning. To evaluate the significance of MRFCD, we contrast the diagnostic results of MRFCD-SSCNN with supervised 1-D CNN upon various labeled data size.

**Figure 8:**
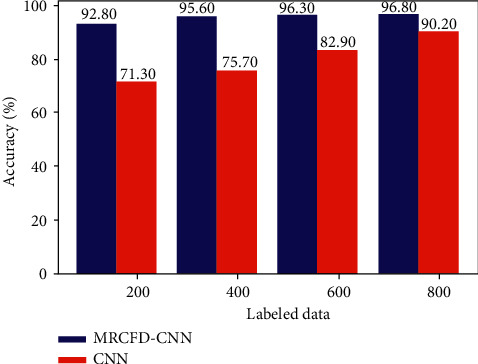
Diagnostic accuracy of MRFCD-SSCNN and CNN.

The semi-supervised model based on MRFCD-CNN is trained by using multiple combinations of labeled and unlabeled samples, in the meantime, the same batch of labeled samples are used to train the supervised model based on CNN. Given the randomness of network architecture, each experiment is executed for 10 times. The average diagnostic results of MRFCD-SSCNN and CNN with various labeled data size are described in Figure 8.

As Figure 8 suggests, the average diagnostic accuracy of CNN trained with 200 labeled samples by supervised learning is only 71.3%, which is nearly 22% lower than that of MRFCD-SSCNN trained with 200 labeled samples and 12600 unlabeled samples. With 800 labeled samples used as the training set, the accuracy of supervised CNN increases from 71.3% to 90.2% while that of MRFCD-SSCNN improved from 92.8% to 96.8%. The gap between the accuracies of the two models with the same amount of labeled data gradually decreases. As the number of labeled samples in the training set changed from 200 to 800, the diagnostic accuracy of MRFCD-SSCNN gradually approaches to 100%. However, the accuracy of CNN is subject to the lack of data with labels in training set, making it difficult to exceed 91%.

Since CNN is a supervised trained deep network that can only use labeled data as training set, the model is prone to over-fitting situations when labeled training samples are lacking. It can be concluded that when MRFCD is constructed and introduced into the diagnostic framework as a semi-supervised learning module, the diagnostic accuracy is greatly improved by making full use of a considerable deal of unlabeled samples to participate in the training process.

#### 4.4.3. The Effect of Hyper-Parameters

In order to evaluate the effect of hyper-parameters to the proposed framework, we executed a set of ablation studies on these hyper-parameters. The results of ablation study on hyper-parameters are shown in Table 5. We compare learning rate in the range of {0.1, 0.01, 0.001}, pool size in the range of {2*∗*2, 3*∗*3, 5*∗*5}, and dropout rate in the range of {0.2, 0.5, 0.7}. The dropout operation improves the generalization of the model by blocking out some of the hidden nodes.

As shown in Table 5, large dropout rate values can lead to the loss of important features, while too small values can lead to more weights. With the chosen hyper-parameters, accuracy of the proposed framework is obviously increased. It indicates that an optimal hyper-parameter value can make the model stability and robustness greatly improved.

In this paper, we choose a deep architecture containing three convolutional layers and three down-sampling layers for feature extraction, and three fully connected layers are utilized for fault classification. The convolutional layer makes up the largest contribution to the model. To evaluate the effect of chosen deep network architecture, we compare the performance and size of models based on various convolutional layers. As shown in Table 6, the accuracies of the models with 2 convolutional layers and 4 convolutional layers are 7.38% and 88.49%, respectively, which are significantly lower than the chosen architecture. It indicates that the model based on 3 convolutional layers can effectively extract representative features from input data and achieve excellent fault recognition. It is worth mentioning that a deeper network structure also leads to increase in the number of parameters as well as increase in the model size.

#### 4.4.4. The Performance Analysis of the Proposed Framework

In order to verify the superiority of the proposed framework, a set of experiments are executed to contrast a variety of typical semi-supervised learning models. In the proposed framework, LapRLS and LapSVM are compared under the same number of labeled samples and unlabeled samples. For SVM-based model, the optimal parameter *C* is searched within the range {10^−5^,10^−4^,…, 10^5^,10^6^} and one-to-all approach is used to solve the learning problem of multi-class data. For LapRLS and LapSVM, the optimal regularization parameters *γ*_*A*_ and *γ*_*I*_ are searched within the range {10^−5^,10^−4^,…, 10^5^,10^6^} using cross-validation method.

To validate the generalization of three semi-supervised learning models trained with various labeled data size, we contrast the learning abilities of these models using the testing set. The classification accuracies and standard deviation of various models are shown in Table 7. As shown in Table 7, learning accuracies of the three models are all improved as the number of labeled samples gradually increased.

Furthermore, the proposed framework based on GAN-MRFCD-SSCNN outperforms LapRLS and LapSVM. Taking the case of using 100 labeled samples, the accuracy of GAN-MRFCD-SSCNN is 7% higher than that of LapRLS. The main reason for performance improvement is that the proposed framework can resolve the misclassification of boundary regions by combining the manifold assumption with the constraints based on both the labeled samples and unlabeled samples. Thus, the smoothness of decision functions for several failure patterns can be improved. However, for LapRLS and LapSVM, the diagnostic accuracies are significantly less optimal, with most of the cases below 90%. The reason is that the multiple classifiers which are used to distinguish between two arbitrary categories are constructed so as to affect the learning results.

#### 4.4.5. Feature Representation Capability

**Figure 9:**
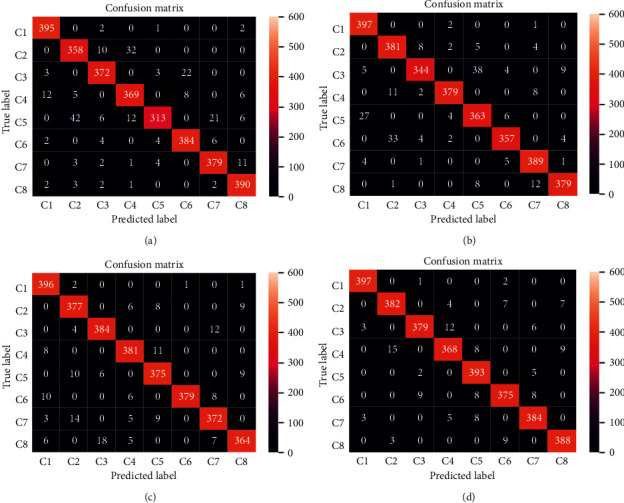
Confusion matrix of each pattern under different labeled samples. (a) size of labeled data: 100. (b) size of labeled data: 200. (c) size of labeled data: 300. (d) size of labeled data: 400.

To further analyze the identification capability of the proposed framework for various failure patterns, the identification accuracies of 8 failure patterns of 3200 testing samples are counted, and the confusion matrix of each failure pattern under different labeled samples are shown in Figure 9. As shown in Figure 9, the health pattern is the most recognizable, and the number of correctly recognized samples is more than other failure patterns. Most importantly, even if the proportion of samples with labels in the training set is very low, the vast majority of failure patterns can be correctly identified with more than 92% accuracy.

**Figure 10:**
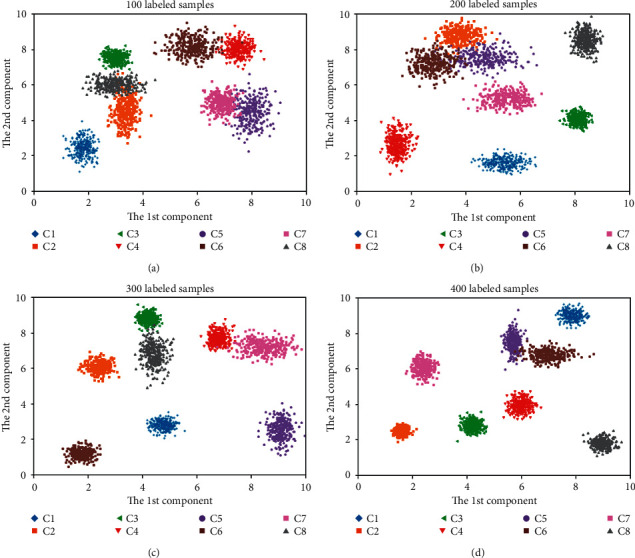
Distributions of each pattern under different labeled samples. (a) size of labeled data: 100 (b) size of labeled data: 200. (c) size of labeled data: 300 (d) size of labeled data: 400.

Due to its powerful feature characterization capability, conventional convolutional neural network has made many achievements in the intelligent machinery fault diagnosis. The proposed framework based on GAN-MRFCD-SSCNN makes full use of CNN to achieve fault feature representation. To visualize the distribution of feature space in an intuitive way, PCA is chosen to actualize dimensionality reduction and take the most important two principal components which can retain 95% original information, and then, exhibit them in a two-dimensional space. Distributions of each pattern under different labeled samples are shown in Figure 10.

As shown in Figure 10, the characteristics of different fault patterns have a better discrimination, particularly when the proportion of data with labels is much higher. The visualization results indicate that the proposed framework can still effectively realize the discrimination and identification of various patterns without sufficient labeled data due to the semi-supervised learning module of MRFCD. The MRFCD module first uses an unsupervised fuzzy clustering method to obtain cluster labels for the entire training set, and then, considers both inter-class and intra-class constraints for the training set.

#### 4.4.6. Runtime Analysis of the Proposed Framework

In order to evaluate the efficiency of the proposed framework, a series of comparative experiments are implemented to analyze the runtime of several supervised learning and semi-supervised learning models. The average runtimes of different diagnostic models with various labeled dataset are shown in Table 8.

As shown as in Table 8, as the number of labeled samples increases, the runtime of each model increases. Since CNN and SVM only use labeled samples for training, they significantly outperform other semi-supervised learning models in training time. However, the weak accuracies of CNN and SVM weaken their advantage in training time.

Comparing the three semi-supervised learning models, the runtime of diagnostic models based on LapSVM and the proposed model is higher than LapRLS. The training time of LapSVM for multi-class datasets is significantly higher than other semi-supervised learning models, and the main reason is that the one-to-many strategy adopted by LapSVM increases the training time during the iterative process. The training time of the proposed model based on MRFCD-SSCNN is the highest, and the reason is that the semi-supervised learning module MRFCD is established on fuzzy clustering and manifold assumptions for the entire dataset. Considering the excellent failure recognition accuracy, the testing time of our model which is nearly 2 seconds is acceptable.

## 5. Conclusions

In this paper, a novel fault diagnostic framework based on generative adversarial network (GAN) and semi-supervised convolutional neural network (CNN) is proposed. There are two typical problems of industrial online monitoring data. The first one is that the amount of normal state data accounts for the vast majority, and the amount of failure patterns data is small. The second problem is that the labeling cost is high, resulting in a great deal of data without labels. Data collected during the operation of the simulation equipment cannot be given corresponding status labels in time. To solve the problem of the lack of typical patterns samples, we utilize GAN to produce data of multiple failure patterns so as to expand the raw data. Additionally, to address the problem of insufficient labeled data, a semi-supervised clustering module based on manifold-regularization-based fuzzy clustering discrimination (MRFCD) is proposed. Then MRFCD is introduced into CNN to construct the pseudo-labels and the loss function of unlabeled data. The semi-supervised diagnostic framework based on GAN-MRFCD-SSCNN is established to achieve intelligent fault diagnosis when the proportion of labeled and unlabeled data is extremely unbalanced.

Based on vibrational signals collected from the actual test rig of automobile main reducer, a range of contrastive experiments are carried out. The contrastive results indicate that GAN, which is regarded as the data expansion module, makes practical efforts on enhancing the accuracy. By considering inter-class and intra-class constraints for the entire dataset and constructing the model based on manifold and clustering assumption, the semi-supervised model based on MRFCD can still obtain better failure identification capability than supervised learning-based model with few labeled samples. Among several classical semi-supervised learning-based models, the proposed framework based on GAN-MRFCD-SSCNN shows superior performance and outperforms the other models by 7% under the same partition of training set.

This research only focuses on intelligent diagnosis of a single fault. However, in actual working conditions, multiple faults may occur simultaneously. In the future, the proposed diagnostic framework can be extended for simultaneous fault diagnosis. Furthermore, the performance of the framework would be improved by applying some novel hyper-parameter optimization methods to CNNs.

## Figures and Tables

**Table 1:** Description of various patterns.

No.	Fault pattern
*C*1	Health
*C*2	Gear burr
*C*3	Gear error
*C*4	Gear hard point
*C*5	Gear tooth broken
*C*6	Gear interference
*C*7	Misalignment
*C*8	Gear crack

**Table 2:** The distribution of dataset.

No.	Fault pattern	Total size	Training set	Testing set	Labeled samples	Unlabeled samples
*C*1	Health	500*∗*1024	400	100	25	375
*C*2	Gear burr	500*∗*1024	400	100	25	375
*C*3	Gear error	500*∗*1024	400	100	25	375
*C*4	Gear hard point	500*∗*1024	400	100	25	375
*C*5	Gear tooth broken	500*∗*1024	400	100	25	375
*C*6	Gear interference	500*∗*1024	400	100	25	375
*C*7	Misalignment	500*∗*1024	400	100	25	375
*C*8	Gear crack	500*∗*1024	400	100	25	375
Total		4000*∗*1024	3200	800	200	3000

**Table 3:** The architecture of the proposed framework.

Purpose	Layers	Number of filters	Kernel size	Output size
*Feature extraction*	Convolutional layer1	16	1*∗*16	1024*∗*16
	Down-sampling layer1	16		512*∗*16
	Convolutional layer2	32	1*∗*6	512*∗*32
	Down-sampling layer2	32		256*∗*32
	Convolutional layer3	64	1*∗*6	256*∗*64
	Down-sampling layer3	64		128*∗*64

*Fault classification*	Fully connected layer1	1	1024	1024
	Fully connected layer2	1	300	300
	Fully connected layer3	1	30	30
	Softmax layer	1		N/A

**Table 4:** Hyper-parameters of the proposed model.

Hyper-parameter	Value
Learning rate	0.001
Dropout rate	0.5
Pool size	1*∗*2

**Table 5:** Ablation study on hyper-parameters.

Hyper-parameters	Values	Accuracy of MRFCD-SSCNN (with 200 labeled samples)
*Learning rate*	0.1	81.2% (±1.33)
	0.01	85.6% (±1.15)
	**0.001**	**93.4% (±0.89)**

*Pool size*	5*∗*5	81.9% (±1.02)
	3*∗*3	87.1% (±1.56)
	**2** *∗ * **2**	**93.4% (±0.89)**

*Dropout rate*	0.2	78.2% (±2.11)
	**0.5**	**93.4% (±0.89)**
	0.7	84.7% (±1.48)

**Table 6:** The performance of various architecture.

Models	Various architecture (with 200 labeled samples)		
	2 convolutional layers	3 convolutional layers	4 convolutional layers
Accuracy	77.38% (±1.59)	93.4% (±0.89)	88.49% (±1.75)
Model size (MB)	0.4	0.5	0.6
GFLOPs	0.006	0.012	0.014

**Table 7:** The accuracies of different diagnostic models.

Labeled data	LapRLS	LapSVM	GAN-MRFCD-SSCNN
100	83.2% (±0.76)	84.7% (±1.21)	92.5% (±0.97)
200	85.9% (±0.93)	86.9% (±0.95)	93.4% (±0.89)
300	87.5% (±1.34)	89.4% (±1.08)	94.6% (±0.93)
400	89.8% (±1.19)	91.1 (±0.77)	95.8% (±1.05)

**Table 8:** Runtime of different diagnostic models.

Labeled data	Models' runtime (s/epoch)	CNN	SVM	LapSVM	LapRLS	MRFCD-SSCNN
100	Training time	3.161	1.246	8.079	6.337	8.136
	Testing time	0.828	0.359	2.091	1.641	2.102

200	Training time	3.209	1.305	8.164	6.425	8.225
	Testing time	0.891	0.395	2.126	1.707	2.172

400	Training time	3.473	1.346	8.217	6.509	8.341
	Testing time	0.913	0.451	2.185	1.726	2.231

## Data Availability

The data were taken from the actual project subject, and readers can contact the author if necessary.
